# Long-Term Mental Health and Quality of Life Outcomes of Neonatal Insults in Kilifi, Kenya

**DOI:** 10.1007/s10578-020-01079-1

**Published:** 2021-01-16

**Authors:** Dorcas N. Magai, Hans M. Koot, Charles R. Newton, Amina Abubakar

**Affiliations:** 1 Department of Clinical, Neuro- and Developmental Psychology, Amsterdam Public Health Research Institute, Vrije Universiteit Amsterdam, Van der Boechorststraat 1, 1081 BT Amsterdam, The Netherlands; 2 Centre for Geographic Medicine Research Coast, Kenya Medical Research Institute, Kilifi, Kenya; 3 Department of Public Health, Pwani University, Kilifi, Kenya; 4 Department of Psychiatry, University of Oxford, Oxford, UK; 5 Institute for Human Development, Aga Khan University, Nairobi, Kenya

**Keywords:** Behaviour, Emotions, Hypoxic-ischemic encephalopathy, Neonatal jaundice

## Abstract

We examined the mental health and quality of life (QoL) outcomes and their correlates of school-aged survivors of neonatal jaundice (NNJ), hypoxic-ischemic encephalopathy (HIE), and a comparison group. The Child Behavior Checklist and the Pediatric Quality of Life Inventory were administered to assess the mental health and QoL of 375 children (134 with NNJ, 107 with HIE, and 134 comparison group) aged 6 to 12 years [Median age 9 (interquartile range 7 to 11)]. The results showed that survivors of NNJ and HIE have mental health problems and QoL similar to the comparison group. Maternal mental health was the predominant covariate of mental health and QoL in survivors of NNJ and HIE. This result could indicate that mothers with mental health problems are more likely to have children with mental health issues, but also that caring for children with these adversities may affect mental health well-being of the caregivers. There is a need for early mental health screening and psychosocial intervention for caregivers and their children to enhance both their mental health and QoL.

## Introduction

Neonatal jaundice (NNJ) and hypoxic-ischemic encephalopathy (HIE) are common problems globally, but are most prevalent in low- and middle-income countries (LMICs) such as those in sub- Saharan Africa (SSA) [1, 2]. NNJ is a result of increased production of bilirubin and reduced excretory capacity of the immature liver of a baby during the first 28 days of life [3]. HIE is characterized by a reduced oxygen or blood flow in the brain before, during, or immediately after birth [4]. Both NNJ and HIE are major causes of brain damage [5–7], leading to both short-term and long-term adverse neurobehavioral outcomes [6, 8].

In high-income countries (HICs), most children survive NNJ and HIE without significant morbidity due to availability of preventive strategies, early diagnosis, and advanced care and treatment. In contrast, in LMICs, especially in rural areas such as Kilifi, about 50% of mothers deliver their babies at home by unskilled birth attendants [9]. Therefore, most children may experience a late diagnosis of NNJ or HIE depending on how soon the caregivers detect illness in their children and how fast they access hospital services. Accessibility to the hospital is further derailed by economic and infrastructural challenges [10–12].

Moreover, survivors of these conditions are likely to be impaired in different developmental domains due to lack of proper guidelines for care and treatment [13], lack of hospital facilities, and inadequate medical equipment and personnel [14]. Given that most families have limited resources, survivors of NNJ and HIE may not be able to receive rehabilitative services which further accentuate the poor long-term outcomes.

Childhood infections are likely to interfere with the development of specific brain regions such as the prefrontal cortex and the subcortical ganglia regions that regulate behaviour and emotions and may develop mental health problems [15] such as emotional and behavioural problems (EBPs). Emotional and behaviour problems manifest as both internalizing problems such as depressive, withdrawn, and anxiety symptoms and externalizing problems like attention problems, aggressive, conduct, and rule-breaking behaviours [16]. Generally, children in SSA and Middle East countries have the highest levels of emotional problems and medium levels of behavioural problems as compared to children from other countries [17]. Specifically, Kenyan children and adolescents are reported to have elevated EBPs in several domains as compared to other children from other countries based on multicultural norms [18].

Although some degree of EBPs are expected in young children, persistent problems [19] may indicate developmental problems which affect the quality of life of the affected children.[17, 18]. Quality of life is a subjective or objective measure of an individual’s well-being, including their physical, social, emotional functioning, as well as their economic status [20]. Health-related quality of life (HRQOL) is a useful measure of general well-being, taking into account the physical, psychological, and the overall impact of health on an individual’s QoL [20]. Despite EBPs and QoL being essential aspects of development, most studies on survivors of NNJ or HIE have focused on the neurocognitive outcomes while the mental health outcomes have received the least attention [21, 22], especially in school-aged children. The few studies on long-term mental health yielded equivocal results. While some reported elevated neurobehavioral problems, mental health disorders, and poor overall functioning of school-aged survivors of NNJ [23–25] and HIE [26–28], other studies did not find differences in mental health outcomes in school-aged survivors of NNJ compared to unaffected children [29, 30].

The long-term outcomes of NNJ and HIE are best understood using dynamic models of human development, such as the bioecological model of human development. According to this model, child development is influenced by both biological and environmental factors to which the child is exposed [31]. A child’s development will be shaped not only by personal attributes and biomedical factors (e.g. neonatal insults, health status, and obstetric factors), but also by psychosocial environments (e.g. family environment, social-economic status, schooling), and the characteristics of his/her caregivers (e.g. caregiver mental health, level of education, and marital status). To identify strategic points of interventions for at-risk children, it is essential to study the relative contribution of each of these factors to the outcomes of survivors of NNJ or HIE.

While the literature presents some data on the mental health outcomes of survivors of NNJ and HIE, most of these studies are based on data from HICs [23–27, 29, 30]. Despite the high burden of NNJ and HIE in SSA, there are no data on the mental health outcomes of school-aged children in this region who survive these conditions. Given the uniqueness of SSA countries, as previously discussed, it is vital to establish the burden of mental health and QoL in children who survived neonatal insults (NNI) in this part of the world. The quality of medical treatment and care, the living circumstances, and the family arrangements in LMICs in SSA may be dramatically different from those in other parts of the world, which raises the question to what extent available scientific knowledge on outcomes of NNI is applicable in SSA countries. Lack of data may impede the availability of needed policies, necessary care and treatment for school-aged survivors of NNJ or HIE who live with some degree of impairment. The limited data on the long-term mental health outcomes of neonatal insults in SSA could be attributed to a lack of research expertise and inadequate access to health care. Additionally, none of the studies has investigated the QoL and correlates of mental health and QoL outcomes of school-aged survivors of NNJ and HIE. In this study, we examined EBPs and QoL of school-aged survivors of NNJ and HIE and the correlates of mental health and QoL outcomes of school-aged survivors of NNJ and HIE born in Kilifi, Kenya.

## Methods

### Study Design

We conducted a cross-sectional study of children aged six to twelve years with a past admission history of NNJ or HIE at Kilifi County Hospital (KCH).

### Study Site

All study procedures and assessments were conducted at the Centre for Geographical Medicine Research-Coast (CGMRC) Neuro-assessment unit situated at the Kenyan Coast. We utilised the Kilifi Health Demographic Surveillance system (KHDSS) [32] to recruit a well-defined cohort who were admitted with severe NNJ or HIE.

### Study Participants

**Fig. 1:**
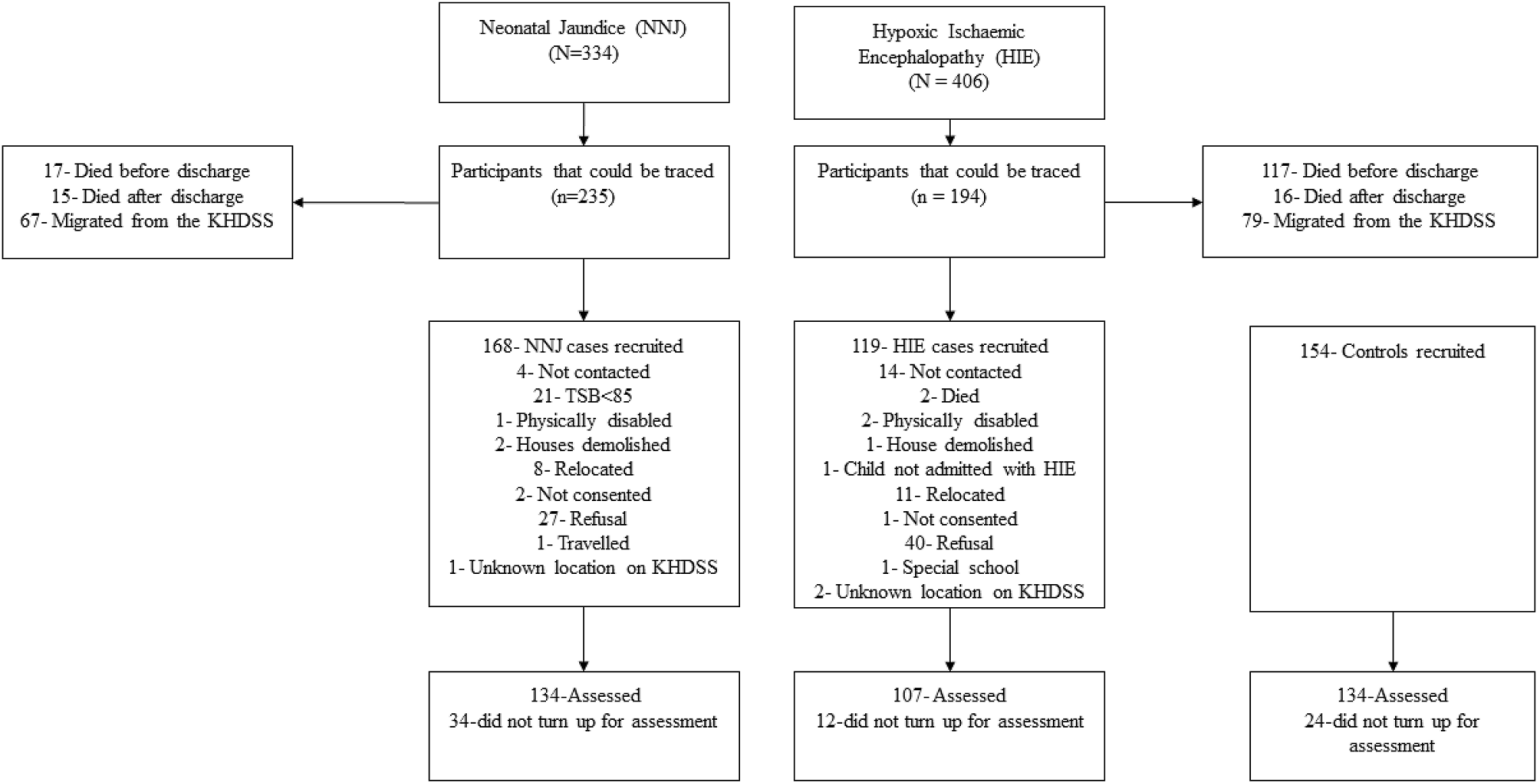
Flow chart of identification, recruitment, and assessments of survivors of neonatal jaundice and hypoxic ischaemic encephalopathy

Children who took part in this study were admitted to KCH in their neonatal period with a diagnosis of either NNJ or HIE. The diagnosis of NNJ was based on clinical laboratory measurement of total serum bilirubin (TSB) as well as medical history and examination during the first 28 days of life. NNJ was defined as a TSB level of > 85 µ/mols/l recorded in the clinical notes. HIE diagnosis was based on the clinical diagnosis recorded by a clinician. HIE diagnosis was given if a child; had convulsions, was unable to breastfeed, had apnoea, and or poor motor tone [33]. The comparison group were identified through the KHDSS and were included in the study if they did not have any history of hospital admission. The children were accompanied to the assessment with their primary caregivers (mostly their mothers). The study reports findings from 375 participants; 134 who survived NNJ, 107 survived HIE, and 134 participants in the comparison group (Fig. 1). The median age of the participants was 9 (interquartile range 7 to 11) years. Of the 375 participants included in this study, 57.3% were males. Thirteen cases had preterm birth (8 survivors of NNJ and 5 survivors of HIE). Most of the caregivers (84%) were married. More than half of the caregivers (69.3%) were Christians, 9.1% were Muslims, while the rest had a traditional religious affiliation. About half of the caregivers (52.5%) had primary education, 34.9% did not have a formal education, while the rest had college or university education. Thirty-nine per cent of the caregivers were farmers, 34.4% were traders, 14.7% were casual labourers, 5.3% were professionals, while the rest had other occupations.

### Study Sample

G-power 3.1 software calculations gave an estimation of 127 participants in the NNJ group [34] and 90 participants in the HIE group [35] to provide a power of 0.95 (*p* = 0.05) to detect medium effect sizes between the affected groups and the comparison group. The number of participants in the comparison group was calculated using frequency matching, where 20 participants were required in each age band (6–12 years).

### Assessments

#### Child-Level Data

##### Child Behavior Checklist (CBCL/6-18)

The CBCL/6-18 [36] was used to collect parent reports about child problem behaviour. The CBCL section on the child’s EBPs contains 118 questions to be answered with: 0 = not applicable, 1 = somewhat or sometimes applicable, and 2 = very much or very often applicable. Item scores are summed into eight syndrome scales, including Anxious/Depressed, Withdrawn/Depressed, Somatic Complaints, Social Problems, Thought Problems, Attention Problems, Rule-breaking Behaviour, and Aggressive Behaviour. These scales can further be combined into an Internalizing (Withdrawn, Somatic Complaints, Anxious/Depressed) and Externalizing (Rule-breaking Behaviour, Aggressive Behaviour) broadband scales, and a Total Problem score can be computed by summing all item scores. The latter three variables were used to compare the mental health outcomes between the affected and the unaffected children and to determine the underlying factors associated with poor mental health outcomes in NNJ or HIE. The Kiswahili version of the CBCL/6-18 has been used in Kenya and provided good psychometric properties with an internal consistency ranging from Cronbach alphas 0.58 to 0.95 depending on the syndrome scale and age group [18]. In this study, the CBCL also had good to excellent internal consistency for the two broadband and Total Problem scales (Cronbach alphas 0.66 to 0.87).

##### Pediatric Quality of Life Inventory (PedsQL)

The PedsQL [37] is a 23-item questionnaire for measuring health-related QoL (HRQL) in children and adolescents ages 2–18 years. In this study, the parent version was completed by the caregiver of the child (6 to 12 years).

The PedsQL has four main scales: physical functioning, emotional functioning, social functioning, and school functioning. Each negatively keyed item is measured with a five-point Likert scale that ranges from “1 = never” to “5 = almost always”. After reverse scoring of the item scores, the scale scores are computed as the sum of the item scores in each scale. These scores are transformed to a 0–100 scale such that higher scores indicate better HRQL.

In this study, a Physical Health Summary Score was computed by summing the reversed individual item scores of the physical functioning scale, and a Psychosocial Health Summary score by computing sum of the items over the number of items answered in the emotional, social, and school functioning scales. A Total Scale Score was computed as the sum of all the items over the number of items answered on all the four scales [38]. The PedsQL 4.0 had already been translated into Kiswahili and used in the Kenyan population, and it had demonstrated a fair to good internal consistency across scales (Cronbach alphas 0.58–0.85) [39].

##### Clinical and Anthropometrical Examinations

A trained clinician conducted a physical examination to determine the motor and sensory neuron responses of the children using a detailed neurological proforma adapted for this study, from a proforma that has been extensively used within the study setting [40].

We did anthropometric assessments and measured the children’s weight, height, middle-upper-arm measurements in centimetres as per the recommendations of the World Health Organization (WHO) [41]. The calculation of height-for-age (HAZ) and weight-for-age (WAZ) was done using the WHO Anthro plus for personal computers version 3.2.2 [42].

##### Medical History

A clinician conducted structured interviews with the caregivers to document the biomedical risk factors. Potential biomedical risk factors included in this study were abnormal pregnancy (defined as post-dated pregnancy, bleeding during pregnancy, pre-eclampsia, or any other health problems during pregnancy), place of birth (home versus hospital), abnormal delivery (defined as postpartum hemorrhage, emergency caesarean section, prolonged labor, obstructed labor, and maternal and fetal distress), delayed crying at birth, breathing problems at birth, hospital admission, presence of febrile seizures, and presence of any other medical problem after discharge from hospital.

#### Demographic Information

Caregivers’ demographic variables that were assessed include sex, age, level of education, marital status, and religion. We also captured information about the child’s sex, age, and number of years of schooling.

##### Caregiver-Level Data

*The Patient Health Questionnaire (PHQ-9)* [43] was administered to assess caregivers mental health in the past two weeks. The PHQ-9 is a 9-item self-report measure with possible scores ranging from 0 to 27. The participant responds to questions ranging from 0 to 3, depending on how well the statement best describes their situation. The PHQ-9 had excellent internal consistencies in this study (Cronbach's alpha = 0.82).

##### Household-Level Data

*The Family Environment Questionnaire (FEQ)* [44] was administered to measure the individual’s perception of their family life. The scale has items that measure the cohesion, expressiveness and conflicts experienced in the relationships in the family; an individual’s personal growth such as independence, moral-religious emphasis; and system maintenance. The items are summed up to obtain a total score. The FES had a relatively low internal consistency in this study (Cronbach’s alpha = 0.50).

*The Kilifi Asset Index* [45] was used to capture the family assets. The tool has items that accounts for different assets owned by the family, including electronic devices, livestock, house and land ownership. The participant is expected to indicate how many assets they own. A total score of assets owned was then computed.

### Statistical Analysis

We compared the demographic characteristics of participants among the three groups (HIE, NNJ, and the comparison group) using analysis of variance (ANOVA) or Chi-square test.

Multivariate analysis of covariance (MANCOVA) was conducted to study group differences on the mental health and QoL measures while adjusting for age, sex, years of education, stunted growth, religion, family asset, maternal education level, marital status, and preterm birth. We conducted univariate regression analysis to identify factors that are associated with the mental health and QOL outcomes. Factors that yielded an association with the *p*-value level ≤ 0.25 were entered in the multiple regression analysis to investigate correlates of the mental health and QoL in NNJ or HIE [46]. We did a stepwise regression analysis with four models adjusting for age, sex, and years of education. In the first model, child characteristics (age, sex, stunted growth, and years of education were entered. In the second model, caregiver and family factors (family asset, maternal lack of education, marital status, family environment, and maternal mental health) were entered. In the third model, obstetric factors (abnormal pregnancy, place of birth, abnormal delivery, hospital admission, crying problems, and feeding problems) were entered, and in the fourth model, medical problems and neurological problems were added.

## Results

Three participants had incomplete demographic information but were included in the analysis. There were significant differences in age, sex, and years of schooling between the survivors of NNJ and the comparison group. No other differences in socio-demographic characteristics among the three groups were observed.

### Mental Health and Quality of Life in Survivors of Neonatal Jaundice and Hypoxic-Ischemic Encephalopathy Versus the Comparison Group

The survivors of NNJ and HIE had slightly higher levels of EBPs but not significantly different from the comparison group in the CBCL scales (Wilks Lambda = 0.97, *p* = 0.860). The results for the three CBCL broad-band scales among the three groups are as follows: Internalizing [F (2, 348) = 1.48, *p* = 0.229]; Externalizing [F (2, 348) = 0.43, *p* = 0.1975]; and Total problems [F (2, 348) = 1.03, *p* = 0.152] (Table 1). There were no significant differences on overall QoL between the affected groups and unaffected group (Wilks Lambda = 0.99, *p* = 0.515). The survivors of NNJ and HIE had higher but not significantly different scores on Psychosocial Health [F (2, 348) = 0.14, *p* = 0.874]; Physical Health [F (2, 348) = 1.60, *p* = 0.203]; and Total Scale [F (2, 348) = 1.38, *p* = 0.253] compared to the comparison group (Table 1).

**Table 1:** Mental health and quality of life in survivors of NNJ, HIE and the comparison group

	NNJ (N = 134)	HIE (N =)	Comparison group (N = 134)	Group differences		
Mental health outcome	Adjusted mean (SE)	Adjusted mean (SD)	Adjusted mean (SE)	*F*	*df*	p-value
Anxious/depressed	2.48 (0.23)	2.83 (0.25)	2.45 (0.23)	0.77	2348	0.465
Withdrawn/depressed	1.93 (0.18)	2.08 (0.200)	1.71 (0.18)	0.97	2348	0.381
Somatic complaints	1.21 (0.17)	1.53 (0.18)	1.21(0.17)	1.07	2348	0.343
Social problems	3.64 (0.26)	3.38 (0.28)	3.31 (0.26)	0.45	2348	0.635
Thought problems	0.53 (0.10)	0.74 (0.11)	0.52 (0.10)	1.36	2348	0.258
Attention problems	2.64 (0.24)	2.95(0.27)	2.35 (0.23)	1.07	2348	0.344
Rule-breaking behaviour	1.61 (0.19)	1.58 (0.21)	1.42 (0.19)	0.26	2348	0.772
Aggressive behaviour	3.29 (0.29)	3.30 (0.32)	2.91 (0.30)	0.41	2348	0.663
Internalizing problems	5.63 (0.43)	6.44 (0.48)	5.37 (0.44)	1.48	2348	0.229
Externalizing problems	4.90 (0.42)	4.65 (0.47)	4.32 (0.43)	0.43	2348	0.649
Total problems	20.34 (1.28)	21.08 (1.42)	18.42 (1.30)	1.03	2348	0.358
Quality of life						
Psychosocial health summary score	70.14 (0.83)	70.68 (0.92)	70.08 (0.85)	0.14	2348	0.874
Physical health summary score	52.34 (1.21)	55.23 (1.34)	52.46 (1.23)	1.60	2348	0.203
Total scale score	61.24 (0.78)	62.96 (0.86)	61.27 (0.79)	1.38	2348	0.253

All outcomes were adjusted for age, sex, years of schooling, middle-upper-arm circumference (muac); stunted growth; religion; family asset; maternal education, marital status, and preterm birth

### Covariates of Mental Health and Quality of Life Outcomes in Neonatal Jaundice

The multiple regression analysis with maternal lack of education, family environment, maternal mental health, and medical problems predicted the Internalizing problems scale [F (7, 133) = 3.38, *R*^*2*^ = *0.17, p* = *0.003*]; however, only maternal mental health had an independent contribution in the regression (β = 0.31, *p* = 0.001). Maternal mental health, neurological, and medical problems predicted Externalizing problems [F (5, 115) = 3.13, *R*^*2*^ = *0.12, p* = *0.011*]. Maternal mental health (β = 0.24, *p* = 0.007) was the only factor with an independent contribution. Family environment, maternal mental health, and delivery problems predicted Total problems [F (5, 122) = 6.88, *R*^*2*^ = *0.22, p* = *0.000*]. Maternal mental health (β = 0.36, *p* = 0.000) remained the only factor contributing independently. Table 2 gives the results of the multiple regression for the three CBCL broad-band scales.

**Table 2:** Correlates of mental health and quality of life in survivors of NNJ

Risk factors	Internalizing problems	Externalizing problems	Total Problems	Psychosocial Health Summary score	Physical Health Summary Score	Total Scale Score
	β (95% CI)	β (95% CI)	β (95% CI)	β (95% CI)	β (95% CI)	β (95% CI)
Step 1 Child factors						
Child’s age in years	–	–	–	–	–	–
Child’s female sex	− 0.09 (− 2.69 to 0.68)	− 0.16 (− 3.33 to 0.19)	− 0.16* (− 9.82 to 0.06)	–	–	–
Stunted growth	− 0.09 (− 2.92 to 0.97)	–	–	–	0.12 (− 1.73 to 8.67)	0.11 (− 1.13 to 5.29)
Years of schooling	− 0.13 (− 0.80 to 0.12)	− 0.17* (− 0.93 to 0.02)	− 0.20* (− 3.01 to − 0.31)	–	–	–
Step 2 Maternal factors						
Family asset	–	–	–	–	–	− 0.08 (− 1.49 to 0.53)
Maternal lack of education	0.12 (− 0.51 to 2.81)	–	–	–	0.11 (− 1.65 to 7.16)	− 0.12 (− 0.87 to 4.76)
Marital status						
Family environment	− 0.10 (− 0.48 to 0.12)	–	− 0.07 (− 1.26 to 0.52)	–	0.18* (0.02 to 1.63)	0.11 (− 0.18 to 0.82)
Maternal mental health	0.18* (0.00–0.4)	0.24** (0.06–0.36)	0.36** (0.53–1.40)	− 0.30** (− 0.71 to − 0.21)	− 0.22* (− 0.88 to − 0.100)	− 0.34** (− 0.72 to − 0.24)
Step 3 Obstetric factors						
Normal delivery (reference)						-
Abnormal pregnancy	–	–	–	–	–	–
Place of birth	–	–	–	–	–	–
Abnormal delivery	–	–	0.03 (− 4.75 to 7.06)	–	0.24* (1.75–12.88)	0.12 (− 0.97 to 5.53)
Hospital Admission				− 0.21* (− 14.31 to − 1.49)	-	− 0.07 (− 8.38 to 3.69)
Crying problems	–	–	–	–	− 0.06 (− 10.58 to 5.04)	− 0.01 (− 4.98 to 4.37)
Feeding problems	–	–	–	–	0.23* (1.35–11.60)	–
Fits	–	–	–	–	–	–
Step 4 Medical problems	0.08 (− 1.56 to 4.14)	0.01 (− 2.82 to 3.18)	–			
Neurological problems	*–*	− 0.07 (− 2.82 to 1.18)	–	0.05 (− 2.37 to 4.33)	0.11 (− 2.04 to 8.24)	0.10 (− 1.41 to 5.09)
R^2^ (P)	0.17 (0.003)	0.12 (0.011)	0.22 (0.000)	0.15 (0.000)	0.19 (0.001)	0.21 (0.001)

Dash line (–) the variable was not carried forward to the multiple regression analysis

*p < 0.05; **p < 0.001;

The multiple regression analysis with maternal mental health, hospital admission, and neurological problems predicted PedsQL Psychosocial Health Summary score [F (3, 127) = 7.20, R^2^ = 0.15, *p* = 0.000], with maternal mental health (β =  − 0.30, *p* = 0.001) and hospital admission (β =  − 0.21, *p* = 0.016) as independent predictive factors. Stunted growth, maternal lack of education, family environment, maternal mental health, abnormal delivery, crying problems, feeding problems, and neurological problems predicted PedsQL Physical Health Summary Score [F (8, 116) = 3.42, R^2^ = 0.19, *p* = 0.001]. Family environment (β = 0.18, *p* = 0.045); maternal mental health (β =  − 0.22, *p* = 0.014); abnormal delivery (β = 0.24, *p* = 0.010), and breast-feeding problems (β = 0.23, *p* = 0.014) were the independently contributing factors. Stunted growth, family asset, maternal lack of education, family environment, maternal mental health, crying problems, hospital admission, and neurological problems predicted Total Scale Score [F (9, 116) = 3.49, R^2^ = 0.21, *p* = 0.001]. Maternal mental health remained the only factor independently associated with Total Scale Score (β =  − 0.34, *p* = 0.000) (Table 2).

### Correlates of Mental Health and Quality of Life Outcomes in Hypoxic-Ischemic Encephalopathy

The multiple regression analysis with family environment, maternal mental health, and presence of fits predicted Internalizing problems [F (3, 92) = 3.67, *R*^*2*^ = *0.11, p* = *0.015*], with presence of fits as the only independent factor (β = 0.21, *p* = 0.045). Stunted growth, family environment, maternal mental health, crying problems, and presence of fits predicted Externalizing problems [F (5, 86) = 5.28, *R*^*2*^ = *0.24, p* = *0.000*]. Maternal mental health (β = 0.26, *p* = 0.011) and presence of fits (β = 0.29, *p* = 0.004) appeared the only independent predictive factors. Stunted growth, family environment, maternal mental health, crying problems, and presence of fits predicted Total problems [F (5, 86) = 6.38, *R*^*2*^ = *0.27, p* = *0.000*]. Maternal mental health (β = 0.27, *p* = 0.008) and presence of fits (β = 0.31, *p* = 0.002) remained as independent predictive factors. Table 3 presents the results of the multiple regression for the three CBCL broad-band scales.

**Table 3:** Correlates of mental health and quality of life outcomes in survivors of HIE

Risk factors	Internalizing problems	Externalizing problems	Total problems	Psychosocial health summary score	Physical health summary score	Total scale score
	β (95% CI)	β (95% CI)	β (95% CI)	β (95% CI)	β (95% CI)	β (95% CI)
Step 1 Child factors						
Child’s age in years	–	–	–	–	–	–
Child’s female sex	–	–	–	–	–	–
Stunted growth	–	0.11 (− 1.0 to 3.61)	0.14 (− 1.54 to 12.21)	0.17 (− 0.57 to 7.96)	0.13 (− 2.56 to 11.30)	0.16 (− 0.99 to 8.46)
Years of schooling	–	–	–	− 0.11 (− 1.72 to 0.46)	–	–
Step 2 Maternal factors						
Family asset	–	–	–	–	− 0.09 (− 3.01 to 1.16)	− 0.07 (− 1.87 to 0.95)
Maternal lack of education	–	–	–	–	− 0.02 (− 7.06 to 5.62)	− 0.04 (− 1.86 to 1.32)
Marital status				0.16 (− 1.05 to 9.16)		0.11 (− 2.71 to 8.41)
Family environment	− 0.09 (− 0.63 to 0.25)	− 0.02 (− 0.45 to 0.36)	− 0.05 (− 1.53 to 0.88)	–	− 0.03 (− 1.36 to 1.01)	–
Maternal mental health	0.17 (− 0.05 to 0.47)	0.26** (0.07–0.55)	0.27** (0.27–1.71)	− 0.33** (− 1.14 to − 0.29)	− 0.04 (− 1.36 to 1.01)	− 0.20* (− 0.91 to − 0.01)
Step 3 Obstetric factors						
Abnormal pregnancy	–	–	–	–	–	–
Place of birth	–	–	–	–	–	–
Abnormal delivery	–	–		–	–	–
Delayed crying	–	− 0.15 (− 4.02 to 0.45)	− 0.14 (− 11.77 to 1.61)	–	–	− 0.05 (− 5.68 to 3.49)
Feeding problems	–	–	–	–	–	–
Hospital admission					− 0.14 (− 19.85 to 4.74)	
Febrile seizures	0.21* (− 0.06 to 5.51)	0.29** (1.21–6.14)	0.31** (4.54–19.30)	0.18 (− 0.04 to 8.92)	–	–
Step 4 Medical problems					− 0.09 (− 17.27 to 7.41)	0.19 (− 0.28 to 9.60)
Neurological problems				0.08 (− 2.59 to 5.92)	0.13 (− 2.24 to 10.83)	0.13 (− 1.70 to 7.21)
R^2^	0.11 (0.015)	0.24 (0.000)	0.27 (0.000)	0.21 (0.002)	0.08 (0.392)	0.14 (0.03)

Dash line (–) the variable was not carried forward to the multiple regression analysis

*p < 0.05; **p < 0.001

Stunted growth, marital status, maternal mental health, hospital admission, presence of fits, and neurological problems predicted PedsQL Psychosocial Health Summary score [F (6, 89) = 3.92, *R*^*2*^ = *0.06, p* = *0.002*], with maternal mental health (β =  − 0.33, *p* = 0.001) as the only independent factor. Stunted growth, family asset, hospital admission, medical and neurological problems did not predict PedsQL Physical Health Summary Score [F (5, 98) = 1.71, *R*^*2*^ = *0.08, p* = *0.139*]. Stunted growth, family asset, maternal lack of education, maternal mental health, hospital admission, medical and neurological problems predicted Total Scale Score [F (6,89) = 2.46, *R*^*2*^ = *0.14, p* = *0.030*], with maternal mental health (β =  − 0.20, *p* = 0.053) as the only independent predictive factor (Table 3).

## Discussion

We investigated mental health outcomes and QoL of school-aged survivors of NNJ and HIE and a community comparison group and the correlates associated with the mental health and QoL outcomes of survivors of NNJ and HIE. The findings indicate that the survivors of NNJ and HIE have comparable EBPs and QoL functioning as the community comparison group. Poor maternal mental health was associated with elevated EBPs in all the three broadband CBCL scales as well as with lowered quality of life in both survivor groups.

The findings of this study indicate that survivors of NNJ or HIE have mental health outcomes comparable to children without neonatal insults. Our results are consistent with the study by Vanborg et al. (2014), who reported that survivors of NNJ were not at an elevated risk of experiencing mental health problems compared to unaffected peers [30]. Similarly, Van Handel et al. (2009) also reported no elevated EBPs in survivors of HIE using the CBCL [35]. These findings, however, contradict other studies which reported more somatic and psychiatric symptoms [24]; attention-deficit disorder [25]; and autism spectrum disorder [23] in survivors of NNJ and elevated neurobehavioral problems in survivors of HIE [26–28].

Various factors may explain our observations. First, earlier studies indicate that Kenyan children in the general population have elevated EBPs (at least two times more in all the syndrome scales) compared to other multicultural standards [18]. Therefore, the comparison group is experiencing higher mental health problems which are not significantly different from the survivors of NNJ and HIE. Second, the interpretation of these results should be understood in the broader perspective of developmental domains such as cognition, executive functions, and memory and the interplay between the individual characteristics and the environment. As children grow older, their brains may compensate for brain injury during the neonatal period—a phenomenon termed brain plasticity [47, 48]. Therefore, impairments reported during early childhood may resolve as children grow older. On the other hand, most of the survivors with severe outcomes may likely have died; therefore, those with severe outcomes may not have survived until school-age. Additionally, most of the neonates in this study did not have severe hyperbilirubinemia; thus, their mental health might not have been affected.

We found no significant differences in the QoL of survivors of NNJ and HIE when compared to healthy children. To the best of our knowledge, there are no studies that have investigated the QoL of school-aged survivors of NNJ and HIE, thus making it difficult for us to interpret the results in the context of earlier findings. However, investigations into the QoL of survivors of NNJ and HIE are vital as health is regarded as the state of complete physical, mental, and social well-being and not merely the absence of disease [49]. Therefore, it is not only essential to understand the health status of individuals but also to understand and improve its quality. The finding that the survivors of NNJ and HIE have comparable QoL is encouraging as this indicates that despite the exposure to the neonatal insults these children can survive, function optimally, and thrive just as well as the unaffected children.

Although there are limited studies on the associations between maternal mental health and outcomes in survivors of NNJ or HIE, our finding that poor maternal mental health is associated with elevated levels of EBPs and lowered QoL in school-aged survivors of NNJ or HIE is consistent with other population-based studies [5, 50–56]. A possible explanation is that there is a complex causal association between the quality of parental care and EBPs outcomes in children. Researchers have suggested that poor maternal mental health may result in weaker attachments to the child and lack of responsiveness to their needs [57]. Higher levels of mother’s negativity may, therefore, exacerbate a child’s poor emotions regulation and non-compliance, which may negatively affect emotional adjustment and externalizing behavioural problems. The finding that poor maternal mental health is associated with lowered QoL is similar to results reported by other studies with different populations [58, 59]. There is a possibility of shared methods variance in that caregivers with poor mental health may report poorer outcomes for their children. The EBP and quality of life data for children was collected from parents; consequently, caregivers with mental health problems perceived their children as presenting with more problems and having a lower quality of life.

Our finding that having seizures during admission was associated with elevated EBPs among survivors of HIE is similar to results in studies which have reported that EBPs are common in children with acute symptomatic seizures [46].

The findings of this study should be cautiously interpreted, given the following limitations. First, the definition of NNJ included children with mild and moderate NNJ and few children had severe NNJ. Second, the mothers of the participants may have suffered recall bias, especially about the medical history of their children at the neonatal stage. Third, we could not perform subgroup analysis based on the severity of HIE as there was limited data on the Apgar score of the children with HIE. Fourth, as we used parental reports to explore the EBPs and QoL in their children, the responses of the parents could include a subjective component and be biased by the mental health state of the caregivers.

Additionally, parents may lack insights into their children’s mental health state. Teachers’ reports could have added more insights into the children’s mental health functioning as the classroom setting may give the teachers a better opportunity to detect mental health problems [60]. Although QoL is regarded as a subjective measure and self-reports are generally preferred [61], a study in the Kenyan population reveal that QoL children’s self-reports and parental reports are inconsistent [39], making it important to include child report of QoL next to parent reports. However, the age of especially the younger children in this study might have hampered the validity of their reports [62]. Lastly, since this is a cross-sectional study, we cannot infer any causal relationships between maternal mental health and children's EBPs. Future studies with longitudinal designs are needed to investigate further and understand this relationship.

## Summary

Neonatal jaundice and HIE are common clinical problems that affect babies during the first days of life and are associated with child mortality and morbidity. Despite the high prevalence of NNJ and HIE in SSA, there is no data on long-term mental health and quality of life outcomes of survivors of NNJ or HIE in SSA. The current study examined the mental health and quality of life outcomes of school-aged survivors of NNJ, HIE, and a comparison group and the correlates of these outcomes in Kilifi, Kenya. We followed 375 participants (134 who survived NNJ, 107 who survived HIE, and 134 unaffected children) aged 6–12 years. We used the CBCL and PedsQL to assess the mental health and quality of life, respectively. Our results suggest that both the survivors of neonatal insults and the unaffected peers have comparably elevated mental health problems and quality of life outcomes. The association of poor maternal mental health to elevated EBPs and lowered QoL suggests a need for early psychosocial and clinical intervention for caregivers and their children to reduce the children’s risk for later development of EBPs and improve functioning in Kenyan children.
